# Comprehensive information management system for a medical research cohort biobank based on quality by design

**DOI:** 10.1186/s12911-023-02318-w

**Published:** 2023-10-16

**Authors:** Lianshuai Zheng, Leilei Wang

**Affiliations:** Biobank of Lianyungang Maternal and Child Health Hospital, Lianyungang Maternal and Child Health Hospital, Jiangsu, China

**Keywords:** Quality by design, Biobank for cohort study, Information construction, Comprehensive information management system

## Abstract

**Background:**

With the development of big health and big data, cohort research has become a medical research hotspot. As an important repository of human genetic resources, biobanks must adapt to the requirements of large-scale and efficient operation. Thus, biobanks urgently need to design and build a legal, convenient, and efficient information management system.

**Methods:**

This study applies the concept of “quality by design” to build a comprehensive biobank information management system based on the analysis of user requirements, legal and regulatory risks, and industry-standard requirements. The system integrates the management of scientific research projects, biological specimens, clinical information, quality control, and multi-dimensional information query and development. After 10 months of its operation, the comprehensive management system was evaluated through statistical analysis of the efficiency of the construction of the pregnancy–birth cohort and the quality of genetic resources.

**Results:**

Since the system’s launch, the statistics on cohort construction efficiency show that the enrollment rate of eligible pregnant women has increased, and the rate of missing volunteers has dropped. The time needed to establish a 1000-person cohort (with complete biological samples and clinical information in early, middle, and late pregnancy) was reduced, and the effective tracking rate of the samples was 77.42%. The error rate of the deep cryogenic refrigerator decreased, with a clinical information integrity rate of 96.47%.

**Conclusions:**

The comprehensive biobank information management system constructed with the “quality by design” concept is well suited to meet the requirements of medical research. This study provides a solution for designing a comprehensive information system for medical institutions’ biobanks.

## Background

Recently, several international biobanks, such as the UK Biobank and Danish National Biobank, have been built, with standardized specimen collection and processing, standardized management, and efficient quality control. This has provided extensive valuable human genetic resources for medical research [1, 2]. In China, the construction of biobanks based on cohort research has developed rapidly. The Beijing Municipal Hepatitis B/AIDS Sample Bank, set up by You’an Hospital, has established a long-term follow-up cohort of hepatitis B and AIDS-related diseases. Its current sample storage capacity of patients exceeds 1 million, and it has accumulated a large number of specimens and information resources for clinical research on hepatitis B and AIDS [3]. Similarly, the Shenzhen National Gene Bank, established and operated by Shenzhen BGI Gene Research Institute, is a national gene bank approved by four ministries and commissions, including the National Development and Reform Commission. To date, nearly 10 million biological samples have been collected and stored. The bank also focuses on data mining and utilization, providing a basic public service platform for life science research and the development of the biotechnology industry in China [4].

As cohort research has become salient in medical research, cohort biobanks must adapt to large-scale and efficient operational requirements. Although many hospitals in China have established their own biobanks, they lack standardized specimen and affiliated clinical information management. The degree of informatization in the process of specimen and clinical information collection, processing, and utilization/destruction is poor [5]. In addition, the quality control systems are incomplete, which easily leads to the loss of precious case samples, poor specimen quality, and incomplete information materials, which then cannot be used for medical research. At the same time, the management of the biobanks is not standardized, which is very likely to lead to violations of laws and regulations, and has a negative impact on the development of the hospital [6].

The management of the cohort biobank is being informatized which involves the management of specimen information stored daily and clinical information related to specimens, which is an important part of the construction of the biobank and the key to the scientific management of the biobank and the scientific utilization of specimen resources [7]. The development of a standardized biobank management system has now become crucial for national hospitals and various scientific research institutions to improve the management of clinical scientific research data [8, 9]. Further, it could become a platform for information engineering. It could play an important role in helping clinical researchers obtain and share important information, standardizing the management of clinical research and biobanks, and laying a foundation to prevent key diseases, with risk factor intervention as the main means (for example, cohort studies on risk factors of birth defects during pregnancy) [10, 11]. Thus, it is essential to tailor a professional and comprehensive information management system according to user requirements [12]. Additionally, with the further strengthening of medical research ethics review and human genetic resource management in China, it could be of great significance to use the biobank management system to record relevant information in the process from human genetic resource collection to use to avoid the risk of violating laws and regulations in the operation of the biobank [13–16].

At present, most biobank management systems are simple sample inventory management systems with single functions, which cannot realize scientific research project management, online administrative approval, quality control management, information sharing, or other functions critical to the standardized construction and management of the cohort biobank [17–19]. In actual use, the system needs to be used together with other systems, such as office automation, and follow-up and scientific research management systems, which greatly increases the investment in management costs. In addition, the collection of basic information and clinical information of specimens usually needs to be manually entered into the system according to the preset fields, which is time-consuming and error-prone. It is understood that the vast majority of biobank management systems on the market are mass-produced, and scientific research users cannot timeously adjust the administrative approval process, workflow, sample field information, and other fixed content according to the needs of research, quickly rendering it obsolescent, causing inconvenience and wastage of money.

**Fig. 1:**
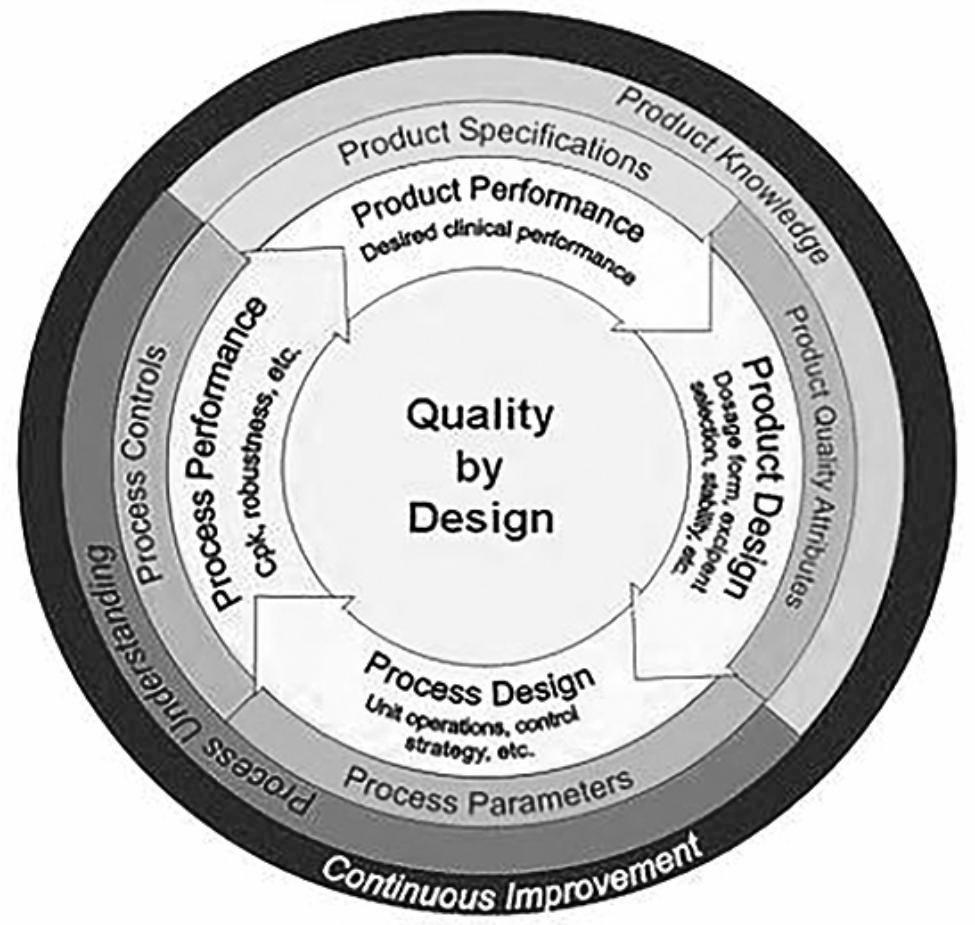
Schematic diagram of the QbD concept

The concept of Quality by Design (QbD) was initially applied to the design and R&D of drugs. After long-term development and the continuous extension of its applications, it has become a systematic R&D method that combines science and technology with quality risk management. It comprehensively applies process analysis technology and risk management to product R&D, analyzes possible technical and legal risks in the quality process and product requirements, conducts process management and quality control from the perspective of overall product R&D, and makes adjustments within the design space to ultimately optimize the design and reduce R&D risks and costs [20]. It provides a new way to build a comprehensive information management system for biobanks (Fig. 1).

Therefore, this study aims to design a legal, convenient, efficient, flexible, and comprehensive biobank management system for medical research using the QbD concept. This can help biobanks control legal and regulatory risks and quality risks, improve the quality and efficiency of cohort establishment, enhance the service capacity of biobanks, better adapt to the requirements of medical researchers, and provide a solution for the design of a comprehensive information system for biobanks.

## Methods

In this study, “Product” means a comprehensive information management system for a medical research cohort biobank, “Process” means the whole process from collection to use of biological samples, “Product Quality Attributes” means the efficiency and quality of cohort establishment, “Process Controls” means key nodes managed during the process from collection to use of biological samples and key parameters affecting their quality attributes (such as pretreatment parameters), “Risks” means legal and regulatory risks/loss of biological samples and information/quality loss, “Design Space” means saving room for the upgrade of the management process and comprehensive information system, such as optimization of project management process and quality control process, and preparation for Laboratory Information System(LIS)/Picture Archiving Communication Systems(PACS)/Jiangsu Provincial Maternal and Child Health Information System /follow-up system interconnection and information sharing.

### Analysis of biobank usage requirements

The design of the comprehensive information management system developed in this study was based on the usage requirements of scientific researchers. This research adopted the format of a conference exchange to investigate and study the ongoing research projects, and proposed applications of various disciplines in the hospital to which the authors are affiliated, and analyzed the specific requirements for specimen collection, processing, storage conditions, and use during the development of research projects. After the meeting, 30 project leaders were asked to answer an open question: “What problem do you want to solve most in the process of using the biobank?” Using their answers, we identified the problems that occur most frequently and that could be solved through an information management system as the starting point of the design. The person in charge of each subject is the potential user of the biobank.

### Legal and regulatory risk analysis

This study investigated relevant laws and regulations on the official website of the Ministry of Science and Technology of the People’s Republic of China, summarized the legal risks in the construction and operation of the biobank, and analyzed the key nodes of quality control through literature retrieval and official website query of the industrial technical specifications and quality certification standards related to the biobank.

### Design and construction of a biobank-integrated information management system

The information management system of biobank for scientific research should be constantly adapted to the regulatory requirements of the state on human genetic resources and the changing requirements of medical research users. At the beginning of the establishment of the information system, various qualifications, certifications, administrative approvals and user requirements for biobanks should be taken into consideration to ensure sufficient design space.

**Fig. 2:**
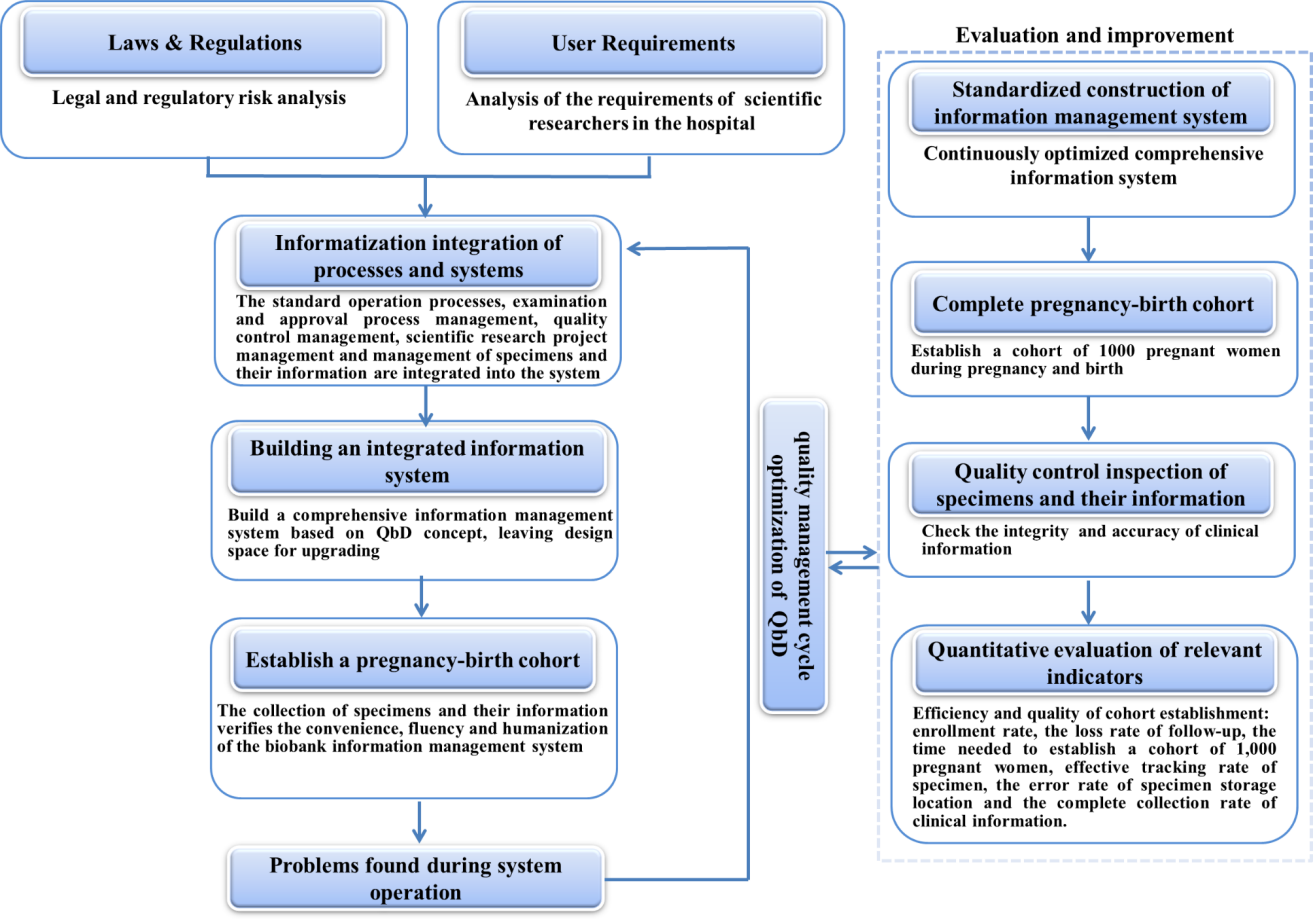
Flow chart of the construction and evaluation of a comprehensive information system for a cohort biobank based on the QbD concept

The design’s process analysis aimed to understand the legal and regulatory risks and the key points of quality control. The design of the standard workflow, management system, and information system was continuously optimized, and a comprehensive information management system was built, covering the entire life cycle of human genetic resources and meeting the requirement of users (Fig. 2).

The system adopts the mature Browser/Server (B/S) architecture and combines with the open source MySQL database for development, which centralises the core part of the system function realisation on the server, simplifying the development, maintenance and use of the system. System users can simply install a browser on their machines. Front-end framework used: Thymeleaf, Bootstrap, Layui; Back-end framework used: Spring Boot, Apache Shiro, MyBatis.The human–computer information interaction was realized by matching the high-speed photographic apparatus and printer.

Optimisation of Big Data Queries: (1) Split the data storage according to certain rules, reduce the size of a single database and table to improve the performance of the database (2). Pre-grouping statistics by day and month to solve the problem of CPU and memory consumption when querying large amounts of data (3). Introduce middleware in the code and upgrade the project by modifying Java DataBase Connectivity(JDBC).

### Evaluation of biobank-integrated information management system

The comprehensive information system designed and built was used to establish a cohort of 1,000 cases of birth defects during pregnancy to evaluate the performance of it according to the efficiency and quality for cohort establishment.

The efficiency and quality of cohort establishment were evaluated by statistical analysis of enrollment rate, the loss rate of follow-up, the time needed to establish a cohort of 1,000 pregnant women, effective tracking rate of specimen, the error rate of specimen storage location and the complete collection rate of clinical information.

### Establishment of pregnancy–birth defect cohort

Pregnant women were recruited into the group at 8–13 weeks of pregnancy to establish a cohort of 1,000 cases of birth defects during pregnancy. Diagnostic information and medical record information of pregnant women at early, middle, and late pregnancy and 42 days of newborn or pregnancy outcome were collected. Peripheral blood at early, middle, and late pregnancy and amniotic fluid, placenta, and umbilical cord/cord blood samples of the birth defects group were collected.

## Results and discussion

### Analysis of usage requirements of the biobank

Our survey of biobank users helped identify the requirements discussed below.

*Improve the efficiency of administrative examination and approval and timely and efficient communication (9 votes)*. Scientific research projects usually need to be approved by the academic committee or ethics committee of the hospital before being carried out in the biobank. This process may involve the scientific research office, academic committee, ethics committee, and other departments. Applicants without relevant experience may lack overall control. There is often a subjective impression of complexity, difficulty, and low speed in applications.

*Quickly and efficiently obtain and record clinical data of biological samples (7 votes).* In the process of using cohort biobanks, the sociodemographic characteristics and clinical information of biobank donors are crucial and usually the basis of grouping and labeling biological samples such as exposure and control group. Therefore, the more detailed the sociodemographic characteristics and clinical data, the higher the value of biological samples. However, the more comprehensive the clinical data to be recorded, the longer the sample registration and warehousing time will be. If the previous stand-alone sample management software or Excel tables are used for management, the manual inputting increases the probability of errors. Therefore, to reduce the registration and entry time and avoid error, some biobankers deposit paper medical records into the storage room. However, as the sample size gradually increases, paper-based information become cumbersome and difficult to retrieve, creating a “dead database.”

*Multi-dimensional information retrieval and invocation (5 votes).* The sample size of a cohort biobank is usually more than 1,000 cases, and may even be hundreds of thousands of cases. The sample types and specifications of cohort biobanks are different, and the clinical information stored or preserved is often complex. Therefore, the sample information and clinical information require multiple retrievals. Traditional methods thus cannot meet the requirements of multi-dimensional and large data processing in prospective and retrospective cohort studies.

*Refined management to ensure the quality of human genetic resources (4 votes).* At present, quality control often focuses on warehousing, but less attention has been paid to certain processing parameters before the storage stage, which can greatly influence the quality of the specimen. These parameters—such as temporary storage time and temperature, warm and cold ischemia time, fixed type and time of tissue samples and the time of separation, the temperature of the temporary storage after separation, the speed, temperature, and time of centrifugation, and the storage-time before centrifugation of the blood samples—play an important role in guiding the quality control of specimens and can save space and improve the quality of specimens. Therefore, to fully guarantee the quality of specimens and more conveniently implement the post-use quality traceability and quality control-related research of specimens, the processing parameters before storage should be properly recorded in the biobank information system.

*Adapt to relevant upgrade requirements and retain upgrade space (design space) (3 votes).* According to the principle of experimental design and data bias, the reliability of medical research conclusions can be rated from high to low as: meta-analysis, randomized controlled trial study, cohort study, medical record control study, and so on. Observational studies such as cohort studies and medical record control studies play a pivotal role in medical research. Additionally, in recent years, big health, big data, and real-world studies have become major research hotspots. Hospitals are also beginning to pay more attention to medical science research. For example, they have increased their focus on data sharing, multi-center multi-role account creation and permission management, docking with other information systems, and allowing for changes in workflow, specimen field information, and processing parameters.

*Fully ensure information security (2 votes).* The security of data stored by traditional stand-alone biobank management software depends on the data generated by manual periodic backup software. Damage to or loss of the host computer or the backup disk poses a serious threat to information security. At present, the mainstream solution is to use cloud backups. Storing the data in multiple different physical or virtual spaces provides greater security.

In response to the above problems and cause analyses, we have developed an integrated information system solution (Table 1).

**Table 1:** User Requirements Analysis and Integrated Information System Solution for Biobanks

Biobank user requirements	solution
Improve the efficiency of administrative examination and approval and timely and efficient communication	A **Project Management** function module is set up in the system to enable online review and approval. The system assigns roles and permissions to different users. Researchers using the biobank can initiate various administrative approvals online and upload the corresponding attached materials, and simply submit the approvals to the biobank administrator. The administrator of the biobank, the director of the department of science and education, and the dean in charge of the hospital can complete the review online. The system is equipped with a notification function. The biobank administrator sends the approval process and various required forms to the researcher’s account, so that users can download and use them at any time and communicate with each other in a timely manner.
Quickly and efficiently obtain and record clinical data of biological samples	A **Specimen Information Management** function module is set up to connect with the HIS system and use the registration number or hospitalization number as the unique identification information to capture the basic information and diagnostic information with one key.
Multi-dimensional information retrieval and invocation	Based on MySQL database, multi-dimensional specimen search types are set up in the system. According to the use of specimens in medical research, the system has set up “query by field”, “batch query (fuzzy query)”, “query by type of specimen”, and “Visual positioning query” function. The system has the functions of exporting and printing the query results. When researchers query the required specimens and affiliated clinical information, they can export all the results to excel table with one click, which is convenient for data analysis of medical research.
Refined management to ensure the quality of human genetic resources	A **Quality Management** function module has been set up in the system to monitor the entire process of human genetic resources from collection to use or destruction, facilitating risk and quality management. According to the project’s research programme, pre-storage handling parameters, processing and dispensing parameters are tied to the appropriate specimen type, prompting staff to reduce manual errors and specimen damage.
Adapt to relevant upgrade requirements and retain upgrade space (design space)	The system is designed in parallel mode for internal and external networks, which can be switched freely. The system is fully prepared for multi-centre research (external network) and open sharing of information across multiple platforms. The **Custom Fields** function is set up in the **System Settings** function module. Users of the system (authorised by the advanced user) can change the approval process, pre-processing parameters of specimens, collection conditions, processing parameters, dispensing parameters, field information of specimens and so on.
Fully ensure information security	The system adopts Browser/Server (B/S) architecture and supports multi-account logins. The servers are installed in a dedicated server room and the operating network is an internal network, which fully guarantees information security. System users can only view and manage specimen and clinical information within their own projects.

### Legal and regulatory risk analysis

Human genetic resources are not only the basis of medical research but also strategic resources related to national security. The Chinese state has always attached great importance to the protection and management of human genetic resources [12–16]. On May 28, 2019, Premier Li Keqiang formally signed the decree of the State Council and issued the Regulations of the People’s Republic of China on the Management of Human Genetic Resources. On May 28, 2020, the Third Session of the 13th National People’s Congress voted to adopt the Civil Code of the People’s Republic of China to provide for and protect the relevant rights of the owners of human genetic resources. On October 17, 2020, “the biosafety law of the People’s Republic of China” was examined and passed by the 13th session of the standing committee of the National People’s Congress. This law has made clear regulations on the collection, preservation, utilization, external supply, and other activities of human genetic and biological resources in China, and has established a legal supervision system for biosafety throughout the chain, which was implemented on April 15, 2021. On July 1, 2023, the Implementing Rules of the Regulations on Human Genetic Resources Management issued by the Ministry of Science and Technology came into force which sets out more detailed requirements for the collection, conservation and use of human genetic resources. At the same time, the Accreditation Criteria for Biobank Quality and Capacity CNAS-CL10:2020, General Requirements for Biobank Quality and Capacity GB/T37864-2019/ISO20387:2018, and the International Association of Biological and Environmental Biobank ISBER should be understood in detail. Likewise, other industrial standards should be understood, as well as general technical requirements such as Technical Specifications for Collection, Preservation and International Cooperation of Human Genetic Resources issued by the Office of Human Genetic Resources of the Ministry of Science and Technology. Biobanks are important places where human genetic resource operations are carried out, the information design of a biobank, such as the information management system, must refer to all the relevant requirements of national laws and regulations on managing human genetic resources, use information means to control the key nodes in the supervision, and avoid potential legal and regulatory risks (Table 2).

**Table 2:** Legal and regulatory risks faced by biobanks and our solution using the Integrated Information Management System

Laws & Regulations/ Industry standards	Risks	Solution
Civil Code of the People’s Republic of China	Strengthening the management and supervision of the collection, preservation, utilisation and external provision of human genetic resources and biological resources in China. The collection, preservation, utilisation and external provision of China’s human genetic resources shall be in accordance with ethical principles and shall not endanger public health, national security or the public interest.	A **Project Management** function module is set up in the system to manage human specimens according to projects. Before using the biobank, the researcher will be assigned an account, fill in the project’s research plan, research content, research purpose, collaborators, the source, type, quantity and purpose of the human genetic resources to be collected online, and then initiate the online approval, which will be reviewed and approved by the administrator of the biobank, the ethical committee of the hospital, the director of the hospital’s science and education department and the dean in charge of the project before carrying out the human genetic resources collection. When human genetic resources are deposited into the repository for preservation, the researcher has to sign an informed consent form with the sample donor, and after uploading it to the system, the staff of the biobank will register and process the information of the donor and the human specimen before depositing them into the repository for preservation. When human genetic resources are released for use or destruction, the researcher fills in the information of the partner online, signs the declaration of compliance with ethical and legal requirements, and then initiates online approval. After review and approval by the administrator of the biobank, the ethical committee of the hospital, the director of the department of science and education of the hospital and the director in charge of the hospital, the staff of the biobank distributes the human genetic resources out of the bank. A **Specimen Information Management** function module is set up to connect with the HIS system and use the registration number or hospitalization number as the unique identification information to capture the basic information and diagnostic information with one key. Simplifying the process of enrolment recruitment and follow-up, reducing their waiting time, increasing their motivation to enrol, and avoiding lost visits and loss of human specimen information and manual entry errors. A **Quality Management** function module has been set up in the system to monitor the entire process of human genetic resources from collection to use or destruction, facilitating risk and quality management. According to the project’s research programme, pre-storage handling parameters, processing and dispensing parameters are tied to the appropriate specimen type, prompting staff to reduce manual errors and specimen damage.
The biosafety law of the People’s Republic of China	The collection of China’s important genetic lineage, specific areas of human genetic resources or the collection of the types and quantities of human genetic resources specified by the competent department of science and technology under the State Council shall be approved by the competent department of science and technology under the State Council. The preservation of China’s human genetic resources; the use of China’s human genetic resources to carry out international scientific research and cooperation; the transport, mailing and carrying of China’s human genetic resources materials out of the country shall be subject to approval. Overseas organisations, individuals and the institutions they set up or actually control to acquire and utilise China’s biological resources shall obtain approval in accordance with the law.	
Implementing Rules of the Regulations on Human Genetic Resources Management	Human genetic resource material means genetic material such as organs, tissues, cells, etc. that contain genetic material such as human genomes and genes. Detailed regulations on the types, quantities and uses of human genetic resources, or on collaborators, research programmes, research content, research objectives, etc.	
Accreditation Criteria for Biobank Quality and Capacity CNAS-CL10:2020	Quality risk control in the collection, preservation, utilisation and destruction of human genetic resources. The main common quality risks in cohort construction include: Loss of donors eligible for enrolment, loss of follow-up, missing sociological information on donors, missing clinical information on human specimens, incorrect storage location in the refrigerator, inappropriate collection and processing methods for human specimens, damage to human specimens	
General Requirements for Biobank Quality and Capacity GB/T37864-2019/ISO20387:2018		
Best Practices for Repositories:Collection, Storage, Retrieval and Distribution of Biological Materials for Research(ISBER)		
Technical Specifications for Collection, Preservation and International Cooperation of Human Genetic Resources		

### Design and construction of biobank-integrated information management system

Based on the QbD concept, we identified the key nodes that affect the quality of a biobank information management system and formulated a design space that could be continuously optimized and upgraded. The system adopts B/S architecture and supports multi-account logins. The server is installed in a dedicated equipment room to fully ensure information security. According to the QbD concept of “Process Controls”, the system was divided into five functional modules: scientific research project management, specimen information management, specimen management, quality control management, and system setting (Table 3).

**Table 3:** Introduction to the functions of the comprehensive information management system

Function module	Overall function	Secondary function classification	Function details
Project Management	Management of scientific research projects to be carried out in biobank	*Examination/Approval	For approval before the warehousing of scientific research projects, users need to upload the research program, Specimen Collection Application Form, Specimen Storage Record Form, Ethical Review Approval, Informed Consent, and other attached materials to initiate online approval, which will be signed and approved by the head of the Scientific Research Office and the hospital leader group.
		Establishment	After project approval is completed, the leader of the biobank shall complete the establishment of the project, enter the name, type, number, funding amount, start and end time, source unit and department, project leader, contact information, proposed storage time of the specimen, special requirements for storage conditions of the specimen, pre-treatment condition of the specimen, and upload the approved relevant materials.
		Conclusion	After the completion of the project, the project leader shall initiate the closing application and explain how to deal with the remaining specimens, such as launching the destruction process or signing the Extensive Informed Consent to continue to preserve the specimens (depending on whether it is signed before the project is carried out).
*Specimen Information Management	*Connect with the HIS system and use the registration number or hospitalization number as the unique identification information to capture the basic information and diagnostic information with one key. Aligned to prepare docking LIS, PACS, and information-sharing Platform	*Socio-demographic Characteristics	The donor’s name, date of birth, ID number, hospital registration number, hospitalization number, telephone number, project, and other basic information are recorded, and the Informed Consent uploaded.
		*Diagnostic Information	All diagnostic information, including diagnosis time and diagnosis content, is captured from the HIS system and can be manually updated at any time.
		*Family Information	Related information of children and spouses can be added, for example, information of children and spouses can be added to the birth defect cohort, which is convenient for the study of genetic diseases in some families.
Specimen Management	Mainly used for the staff of the biobank to classify, store, retrieve, outbound and return the processed specimens	Space Management	The space management of the deep low temperature refrigerator, liquid nitrogen tank, wax block cabinet and other storage equipment and the storage space allocation of projects.
		Specimen Collection	The types of specimens collected are mainly from birth defects, and included other types of specimens, which are divided into blood, tissue, and body fluids. The second level is divided into peripheral blood, umbilical cord blood, umbilical cord, placenta, fetal membrane, tumor tissue, adjacent tissue, normal tissue, amniotic fluid, urine, alveolar lavage fluid, and vaginal secretions. The third level is divided into serum, plasma, whole blood, maternal placenta, fetal placenta, intermediate placenta, amniotic fluid supernatant, and amniotic fluid precipitation. The fourth layer is all kinds of cells. The fifth layer is DNA, RNA, and protein.
		Specimen Reception	After processing and packaging, the specimens are associated with the corresponding donors and the corresponding projects, and labeled in accordance with the sequence and coding rules, and then put into boxes, shelves, and storage. The label is designed to be encrypted according to the coding rules, and no patient privacy information appears.
		Inventory Retrieval	Mainly used to query the sample type, quantity, location, and donors before stock out.
		Specimen Distribution	Mainly used for ex-warehouse of specimens when they are used or destroyed. It is required to submit the Application Form for Ex-warehouse or Destruction of Specimens, the Letter of Commitment for Use of Specimens and other attachment materials and to initiate the ex-warehouse application.
		Specimen Return	The restorage of the remaining specimens after use, and automatically recording the number of freeze/thaws.
*Quality Management	Management of all processes including project approval and the quality control of the life-cycle management of specimens and their information including collection, preservation, output records, utilization, or destruction.	*Data Query	Includes inquiry functions such as examination and inquiries on approval records, specimen information, pre-treatment condition, receipt/issue/return record.
		*QC Records	Mainly used for quality control personnel to upload quality control schemes and overall quality control results including the date of quality control, sample information, related pictures (DNA/RNA/ protein electrophoresis pictures, etc.).
		*Destruction Record	Used for quality control personnel to record the types, quantities, items, reasons for destruction, methods of destruction, and related picture records of specimens destroyed.
		*Account Log	All account operations will be recorded for quality and safety traceability of specimens and their information.
System Settings	Account and Operational Limits of Authority management of the biobank management system, and the custom Settings in the system can be changed freely according to the actual usage requirements, which fully reflects the design space concept of QbD	Account Creation	Create a user account and password.
		Role Assignment	Assign roles to new accounts and set different permissions for each role. The head of the scientific research office and the leader of the branch in charge of the hospital have the permission to sign and approve, view the scientific research projects in the database, collection statistics of specimens, and so on. The head of the biobank has authority for partial approval, scientific research project management, specimen information management, specimen management, system setting, and quality control management. The biobank information administrator has authority for specimen information management, specimen management, handling the notification of entry application and exit application, and so on. The specimen administrator of the biobank has the permission for specimen management. The quality control manager of the biobank has authority for quality control management. The project leader has the authority to initiate project approval, project conclusion, donor information inquiry, specimen entry application, inventory retrieval, specimen exit application, and return application.
		*Custom Fields	Customize settings or adding comments for some special specimen types, storage schemes, processing capacity, and so on. The approval process can be changed under specific circumstances.

The function marked with “*” is unique to this comprehensive information system, which is used to solve the problems existing in the current biobank management, meet the use needs of scientific researchers in medical institutions, and avoid the risks of relevant laws and regulations

**Fig. 3:**
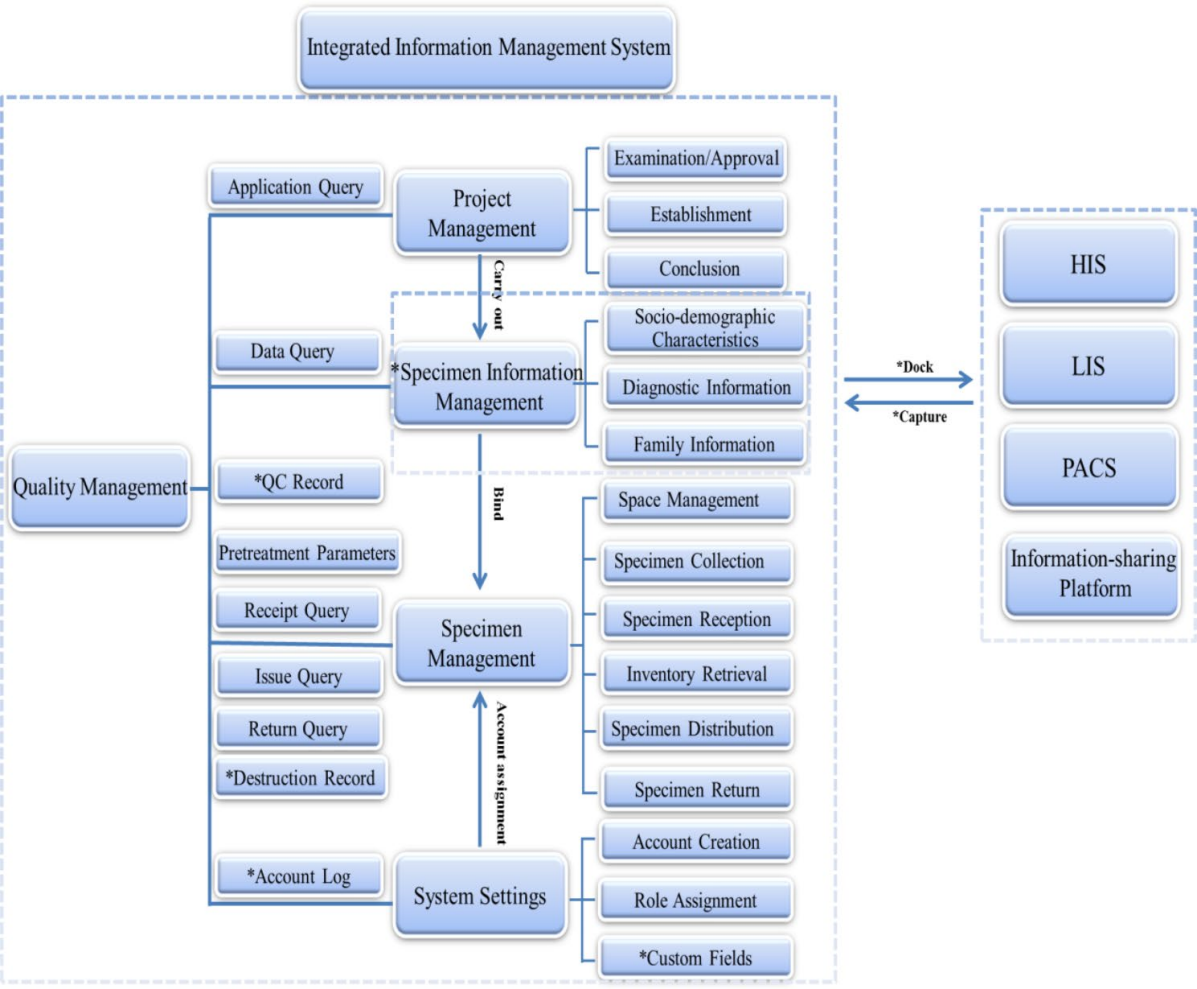
Overall architecture planning of the cohort biobank information management system The function marked with “*” is unique to this comprehensive information system, which is used to solve the problems existing in the current biobank management, meet the use needs of scientific researchers in medical institutions, and avoid the risks of relevant laws and regulations

Compared with other existing information management systems for biobanks, our comprehensive information management system has the following innovations (Fig. 3). (1) The approval process for the collection, preservation, utilization, and destruction of human genetic resources, including relevant annex materials such as electronic ethical review approval and informed consent, was implemented for online approval in project and process management. (2) Interfacing with HIS, real-time capture of sociological information, diagnostic information, and medical record information to simplify the process of donor recruitment during the establishment of the cohort; automatic tagging of donors in the cohort, and reminding of specimen collection and questionnaire completion during readmission. (3) The quality control parameters in the system were recorded, especially the sample pre-processing parameters, as well as the system operation logs of the processing and warehousing operators, to facilitate quality tracing at a later stage. The system is equipped with processing parameter settings and operation prompts throughout the entire life cycle of human genetic resources from collection to use. (4) The system is designed in parallel mode for internal and external networks, which can be switched freely. The system is fully prepared for multi-centre research (external network) and open sharing of information across multiple platforms. With super administrator authorisation, the project approval process, specimen field information, and human genetic resource processing parameters can be altered in response to changes in workflow and research protocols.

### Evaluation of biobank-integrated information management system

A cohort of 1,000 cases of birth defects in a pregnancy–birth population was established to check whether the biobank system we designed could meet the requirements of daily work, is convenient to use, and runs smoothly.

Statistical analysis was conducted on the efficiency of cohort establishment, quality control and detection of biological samples, and the quality of their information since the launch of the comprehensive information management system. The results show that the enrollment rate of eligible pregnant women increased from 9.40% at the commencement of the study to 28.81% at the end of the ten months, the average daily enrollment rate increased from 6.2 to 12.6 per day, the loss rate of follow-up decreased from 13.33 to 4.76%, and the time needed to establish a cohort of 1,000 pregnant women (complete biological samples and clinical information in the first, second, and third trimesters) was shortened by 33.33%. Among the 1,420 eligible pregnant women recruited in the 10 months since the launch of the system, 31 cases of defect outcome were tracked, and 24 cases of defect specimens including peripheral blood, placenta, umbilical cord, and umbilical cord blood, were collected, with an effective tracking rate of 77.42%. The comprehensive management system adopts a lock-space placement mode and a two-step review and storage method, which reduces the error rate of specimen storage location from 5.00 to 0.51% in a -80℃ deep cryogenic refrigerator. With the docking of HIS, the clinical data functions such as social demographic information, outpatient diagnosis information, and grade-5 electronic medical records can be captured with one click, and the complete collection rate of clinical information is 96.47%.

Our system, based on the QbD concept and the usage requirements of scientific researchers, considers all aspects, from the overall planning of the workflow established by the cohort biobank to the management of the key nodes of quality control by information means. As demonstrated here, the system can effectively improve the efficiency of the establishment of the cohort, ensure the quality and quantity of biological samples for medical research, improve the level of medical research, adapt to the national regulatory requirements for human genetic resources, and improve the standardized construction and management of biobanks.

## Conclusions

The design and development of an comprehensive information system for biobanks is a complex task involving many industries, and the difficulty lies in how to take into account the requirements of scientific research users and the operation management requirements of biobanks and “translate” them into the requirements for the computer system operation, and accurately transfer them to the programmers to help them build a more suitable system. This will help programmers to build a more suitable system and shorten the development cycle. This study analyses the basic elements of the construction of an integrated information system for biobanks using the QbD concept, provides a construction plan for system developers, and offers a solution for the management of medical research biobanks.

## Data Availability

The datasets used and/or analysed during the current study are available from the corresponding author on reasonable request.

## Abbreviations

BIMS: Biobank Information Management System
QbD: Quality by Design
JDBC: Java DataBase Connectivity
HIS: Hospital Information System
LIS: Laboratory Information System
PACS: Picture Archiving Communication Systems
